# Impact of Tumor Burden on Normal Organ Distribution in Patients Imaged with CXCR4-Targeted [^68^Ga]Ga-PentixaFor PET/CT

**DOI:** 10.1007/s11307-022-01717-1

**Published:** 2022-03-21

**Authors:** Sebastian E. Serfling, Constantin Lapa, Niklas Dreher, Philipp E. Hartrampf, Steven P. Rowe, Takahiro Higuchi, Andreas Schirbel, Alexander Weich, Stefanie Hahner, Martin Fassnacht, Andreas K. Buck, Rudolf A. Werner

**Affiliations:** 1 Department of Nuclear Medicine, University Hospital Würzburg, Oberdürrbacherstr. 6, Würzburg, 97080 Germany; 2 Nuclear Medicine, Medical Faculty, University of Augsburg, Augsburg, Germany; 3 The Russell H Morgan Department of Radiology and Radiological Sciences, Johns Hopkins School of Medicine, Baltimore, MD USA; 4 Okayama Graduate School of Medicine, Dentistry and Pharmaceutical Sciences, Okayama University, Okayama, Japan; 5 Department of Internal Medicine II, Gastroenterology, University Hospital Würzburg, Würzburg, Germany; 6 Division of Endocrinology and Diabetes, Department of Medicine I, University Hospital Würzburg, Würzburg, Germany

**Keywords:** CXCR4, C-X-C motif chemokine receptor 4, PET, [^68^Ga]PentixaFor, [^177^Lu]/[^90^Y]PentixaTher, Theranostics, Endoradiotherapy

## Abstract

**Background:**

CXCR4-directed positron emission tomography/computed tomography (PET/CT) has been used as a diagnostic tool in patients with solid tumors. We aimed to determine a potential correlation between tumor burden and radiotracer accumulation in normal organs.

**Methods:**

Ninety patients with histologically proven solid cancers underwent CXCR4-targeted [^68^Ga]Ga-PentixaFor PET/CT. Volumes of interest (VOIs) were placed in normal organs (heart, liver, spleen, bone marrow, and kidneys) and tumor lesions. Mean standardized uptake values (SUV_mean_) for normal organs were determined. For CXCR4-positive tumor burden, maximum SUV (SUV_max_), tumor volume (TV), and fractional tumor activity (FTA, defined as SUV_mean_ x TV), were calculated. We used a Spearman's rank correlation coefficient (ρ) to derive correlative indices between normal organ uptake and tumor burden.

**Results:**

Median SUV_mean_ in unaffected organs was 5.2 for the spleen (range, 2.44 – 10.55), 3.27 for the kidneys (range, 1.52 – 17.4), followed by bone marrow (1.76, range, 0.84 – 3.98), heart (1.66, range, 0.88 – 2.89), and liver (1.28, range, 0.73 – 2.45). No significant correlation between SUV_max_ in tumor lesions (ρ ≤ 0.189, *P* ≥ 0.07), TV (ρ ≥ -0.204, *P* ≥ 0.06) or FTA (ρ ≥ -0.142, *P* ≥ 0.18) with the investigated organs was found.

**Conclusions:**

In patients with solid tumors imaged with [^68^Ga]Ga-PentixaFor PET/CT, no relevant tumor sink effect was noted. This observation may be of relevance for therapies with radioactive and non-radioactive CXCR4-directed drugs, as with increasing tumor burden, the dose to normal organs may remain unchanged.

## INTRODUCTION

C-X-C motif chemokine receptor 4 (CXCR4) is involved in migration of tumor cells and angiogenesis in various solid cancers [1]. High expression of CXCR4 allows its targeting and visualization using the positron-emitting radiotracer [^68^Ga]Ga-PentixaFor [2]. CXCR4-directed molecular imaging has been applied in various clinical scenarios, including multiple myeloma (MM) [3], lymphoma [4], and also in solid tumors [5, 6]. Beyond imaging, the theranostic equivalent [^177^Lu]/[^90^Y]-CXCR4 (PentixaTher) has also been administered, e.g., as antitumor therapy or to achieve bone marrow ablation followed by conditioning therapy [7, 8]. In addition, CXCR4-directed PET may also be used to quantify the target in vivo prior to initiation of therapy with agents such as the CXCR4 inhibitor Plerixafor [9, 10] or endoradiotherapy with [^177^Lu]Lu-/[^90^Y]Y-PentixaTher [7, 8].

The uptake of a radiopharmaceutical in tumor lesions and normal organs, however, is influenced by various clinical variables. For instance, a decrease in radiotracer accumulation in normal organs in patients with high tumor burden has been reported for theranostic targets other than CXCR4 [11], e.g., for somatostatin receptor (SSTR) and prostate specific membrane antigen (PSMA)-targeting radiotracers [11, 12]. In this regard, such a tumor sink effect may then also have a significant impact on both radiolabeled and non-radiolabeled CXCR4-targeted therapies. Administered activity of [^177^Lu]Lu-/[^90^Y]Y-PentixaTher or drug doses of “cold” CXCR4 antagonists could then be increased in patients with high tumor burden, maximizing efficacy in sites of disease and reducing off-target effects in unaffected organs. In this study, we aimed to identify a potential tumor sink effect in a cohort of patients with solid tumors who underwent CXCR4-directed [^68^Ga]Ga-PentixaFor PET.

## MATERIALS and METHODS

In this retrospective study, we included 90 patients with solid cancers that were all imaged with [^68^Ga]Ga-PentixaFor PET/CT. Three patients were scanned twice, and thus, a total of 93 scans were included. Parts of this cohort have also been described in [5, 13–15], however without investigating a tumor sink effect. Individuals were most commonly diagnosed with adrenocortical carcinoma (29/90, 32.2%) and neuroendocrine neoplasms (NEN, 22/90, 24.4%). In the remaining cohort, diagnoses were small cell lung cancer (12/90, 13.3%), non-small cell lung cancer (7/90, 7.8%), and pancreatic cancer (5/90, 5.6%). Additional solid tumors included head and neck cancer, liver carcinoma, cholangiocarcinoma, renal cell carcinoma, ovarian carcinoma, pleural mesothelioma, osteosarcoma, and mediastinal tumor (15/90, 16.7%; Table 1). All patients signed written informed consent for diagnostic tests. The institutional review board at the University of Würzburg waived the need for further approval due to the retrospective nature of this study (No. 20210726 02).

**Table 1.** Patients’ characteristics. For age, mean ± standard deviation is displayed. Percentages are given in parentheses. ACC = adrenocortical carcinoma, NEN = neuroendocrine neoplasms, SCLC = small cell lung cancer, NSCLC = non-small cell lung cancer. *includes head and neck cancer (n = 4), liver carcinoma (n = 4), cholangiocarcinoma (n = 2), renal cell carcinoma, ovarian carcinoma, pleural mesothelioma, osteosarcoma, and mediastinal tumor (n = 1, respectively)

Clinical Variable		Number of Patients
Tumor entity	ACC NEN	29/90 (32.2) 22/90 (24.4)
	Other*	15/90 (16.7)
	SCLC	12/90 (13.3)
	NSCLC Pancreatic Cancer	7/90 (7.8) 5/90 (5.6)
Age (in years)	59.6 ± 12.6	
Female		41/90 (45.6)

*Imaging Procedure.* [^68^Ga]Ga-PentixaFor PET/CT was carried out using a Siemens Biograph mCT (64 or 128, Siemens Healthineers, Erlangen, Germany). We conducted scans from the vertex of the skull to the proximal thighs approximately 60 min after injection of 137 MBq (median; range, 64 – 164) [^68^Ga]Ga-PentixaFor. CT with and without contrast enhancement was also performed, and PET images were reconstructed as implemented by Siemens Esoft (Siemens Healthineers, Erlangen, Germany) [5].

*Image Analysis.* Images were analyzed by a single reader (ND) and verified by two experienced readers (SES and RAW). For normal biodistribution of [^68^Ga]Ga-PentixaFor, radiotracer accumulation has been described in the heart, liver, spleen, bone marrow, and kidneys [8, 16]. As such, volumes of interest (VOIs) were placed covering those organs as previously described in [17]. For assessing unaffected bone marrow, we used the average from three VOIs (placed in the cervical (C2), thoracic (Th7), and lumbar region (L5), respectively). As such, to assess normal organ uptake, a total of 8 VOIs were placed for every patient. Moreover, the tumor burden was also manually segmented by placing up to three VOIs for every organ compartment. VOIs were defined as most intense in uptake and with largest diameter, thereby avoiding a partial volume effect [18]. The following organ compartments were included: primary tumor site, skeleton, lymph nodes, liver, lung, and soft tissues. To assess uptake in normal organs, mean standardized uptake values (SUV_mean_) were recorded [19]. For the tumor burden, average maximum SUV (SUV_max_), sum of tumor volume (TV, in cm^3^), and fractional tumor activity (FTA, defined as SUV_mean_ x TV) were calculated, as described in [19].

*Statistical Analysis.* GraphPad Prism version 9.3.1 (GraphPad Prism Software, La Jolla, CA, USA) was used for statistical analyses. A Spearman's rank correlation coefficient (ρ) was determined to investigate correlations between normal organ uptake and tumor burden. A *P*-value of < 0.05 was considered statistically significant.

## RESULTS

*Quantitative Assessment in Normal Organs and Tumor Burden.* Thirteen patients had undergone nephrectomy, and four patients had prior splenectomy. In three subjects, normal hepatic uptake could not be identified due to extensive metastatic involvement of the liver. In another two patients, the respective three VOIs per patient in the bone marrow could not be placed due to widespread disease in the skeleton. As such, a total of (13 + 4 + 3 + 6 =) 26 normal organ VOIs were not drawn and those organs were excluded from further analysis. The overall number of VOIs placed on organs was therefore as follows: (8 VOIs/per patient × 93 scans) – 26 VOIs = 718 VOIs. The median SUV_mean_ in unaffected organs was 5.2 for the spleen (range, 2.44 – 10.55), 3.27 for each the left and right kidney (range, 1.52 – 17.4), followed by bone marrow (1.76, range, 0.84 – 3.98), heart (1.66, range, 0.88 – 2.89), and liver (1.28, 0.73 – 2.45). For assessing the tumor burden, a total of 405 VOIs were placed (median four per scan). The distribution of lesions among organ compartments was as follows: lymph nodes 108/405 (26.7%), liver lesions 96/405 (23.7%), soft tissue lesions 64/405 (15.8%), primary tumors 52/405 (12.8%), lung lesions 45/405 (11.1%), and skeleton 40/405 (9.9%). Median SUV_max_ was 7.8 (range, 3.98 – 22.02), and median TV was 44.12 (range, 1.91 – 817.2). For FTA, we recorded a median of 259.2 (range, 7.77 – 7910.2; Table 2).

**Table 2.** Uptake in normal organs and tumor lesions. For normal organs, mean standardized uptake value was used. *Mean and standard deviation (SD) is only presented for normally distributed data. R = right. L = left. SUV_max_ = maximum standardized uptake value, TV = tumor volume, FTA = fractional tumor activity (mean standardized uptake value x TV)

		Parameter	Minimum	Median	Maximum	Mean*	SD*
**Normal Organs**	Heart		0.88	1.66	2.89	1.71	0.73
	Liver		0.73	1.28	2.45	–	–
	Spleen		2.44	5.2	10.55	–	–
	Bone Marrow		0.84	1.76	3.98	–	–
	Kidney R		1.67	3.27	17.4	–	–
	Kidney L		1.52	3.27	13.46	–	–
**Tumor Burden**		SUV_max_	3.98	7.8	22.02	–	–
		TV	1.91	44.12	817.2	–	–
		FTA	7.77	259.2	7910.2	–	–

**Fig. 1.**
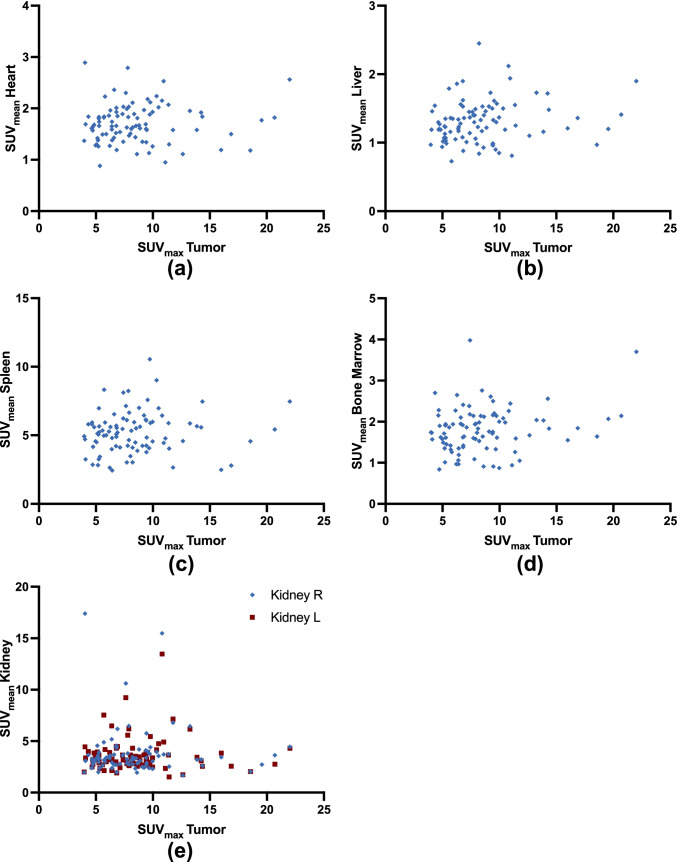
**Correlations of mean standardized uptake values (SUV**_**mean**_**) from organs (a, heart; b, liver; c, spleen; d, bone marrow; and e, kidney) with tumor-derived maximum standardized uptake values (SUV**_**max**_**).** Rhombuses and squares are partially overlaid. No significance was reached. R = right. L = left

**Fig. 2.**
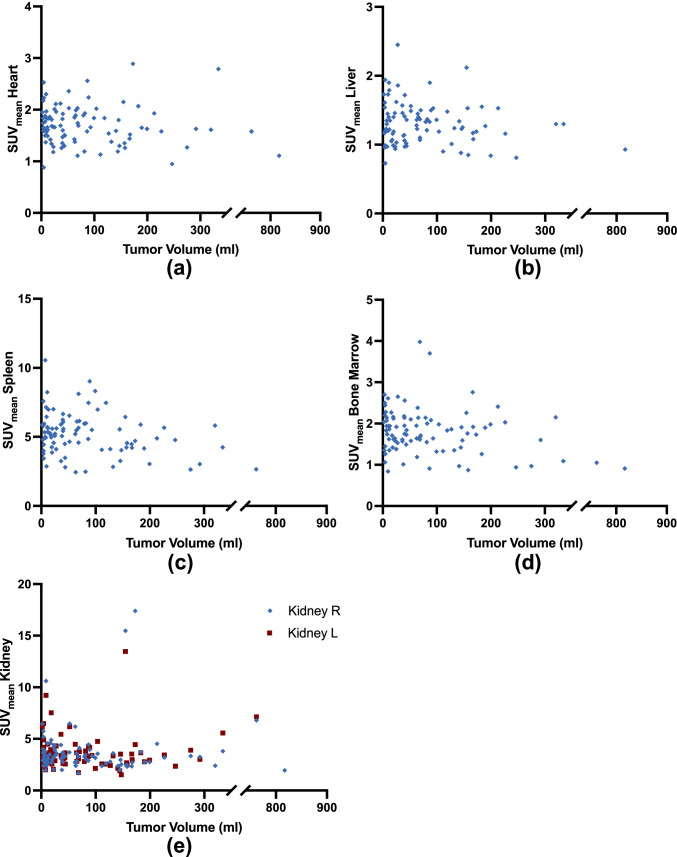
**Correlations of mean standardized uptake values (SUV**_**mean**_**) from organs (a, heart; b, liver; c, spleen; d, bone marrow; and e, kidney) with PET-based tumor volume (cm**^**3**^**).** No significance was reached. Rhombuses and squares are partially overlaid. R = right. L = left

**Fig. 3.**
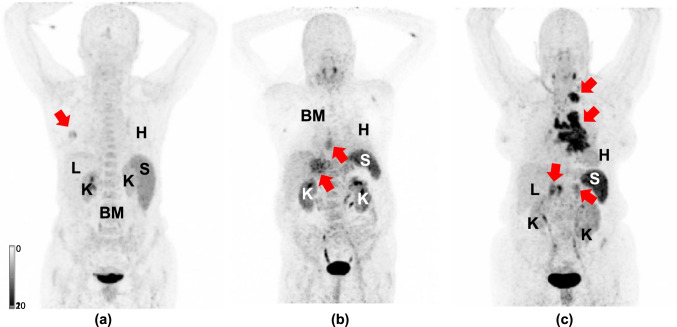
**Planar maximum intensity projection (MIP) of patients with different tumor burden which have been scanned using [**^**68**^**Ga]Ga-PentixaFor PET.** Patient in (a) has low, patient in (b) has intermediate and patient in (c) has high tumor burden. Red arrows indicate tumor lesions. On a visual assessment, normal organ uptake did not decrease in patients with high tumor burden. Due to the extensive tumor burden in (c), bone marrow (BM) was not marked. H = heart, L = liver, K = kidney, and S = spleen

*Correlation of Normal Organ Uptake with Tumor Burden.* Table 3 provides an overview of all correlations. There was no significant correlation between SUV_max_ (ρ ≤ 0.189, *P* ≥ 0.07; Fig. 1), TV (ρ ≥ -0.204, *P* ≥ 0.06; Fig. 2) or FTA (ρ ≥ -0.142, *P* ≥ 0.18) with organ uptake. Figure 3 displays three individuals with different amount of tumor burden on CXCR4-directed PET. On a visual assessment, radiotracer accumulation in normal organs did not appear to decrease in patients with increased tumor burden.

**Table 3.** Spearman’s Rho for correlations between organ uptake and tumor burden. For normal organs, mean standardized uptake value (SUV_mean_) was used. SUV_max_ = maximum standardized uptake value, TV = tumor volume. FTA = fractional tumor activity, defined as SUV_mean_ x TV. ρ = Spearman’s Rho. R = right. L = left

			Tumor Burden		
			SUV_max_	TV	FTA
**Normal Organs**	Heart	ρ *P*	0.084 0.42	-0.141 0.18	-0.105 0.32
	Liver	ρ *P*	0.189 0.07	-0.092 0.39	-0.021 0.85
	Spleen	ρ *P*	0.126 0.24	-0.204 0.06	-0.128 0.23
	Bone Marrow	ρ *P*	0.16 0.13	-0.199 0.06	-0.112 0.29
	Kidney R	ρ *P*	0.05 0.64	-0.168 0.12	-0.142 0.18
	Kidney L	ρ *P*	-0.015 0.89	-0.112 0.31	-0.097 0.38

## DISCUSSION

[^68^Ga]Ga-PentixaFor has been utilized for CXCR4-directed molecular imaging of patients with multiple myeloma or lymphoma [4, 20, 21], but also in various solid tumors [5, 6, 22]. In this study, we evaluated the biodistribution of this agent in normal organs and tumor lesions and did not observe a relevant tumor sink effect. Those findings may be relevant for therapy with radioactive or non-radioactive CXCR4-directed drugs, as with increasing tumor burden, the dose to normal organs may remain unchanged.

For theranostic radiotracers such as the SSTR-directed agent [^68^Ga]Ga-DOTATATE, Beauregard and coworkers reported on a decrease of radiotracer accumulation in normal organs in patients with advanced disease. As such, they recommended modifying the therapeutic activity of the ß-emitting analog, [^177^Lu]Lu-DOTATATE [11]. These findings, however, were in contrast to investigations using the SSTR-targeted radiotracer [^68^Ga]Ga-DOTATOC, where no substantial tumor sink effect was observed [17], possibly due to differences in binding affinity to the target [23].

For PSMA-directed imaging, a relevant tumor sink effect was recently observed in a multi-center study. The authors concluded that PSMA-directed radioligand therapy could be safely conducted with increasing activity, irrespective of the tumor burden [12]. In this study, we aimed to elucidate whether a tumor sink effect may also occur in patients imaged with [^68^Ga]Ga-PentixaFor. In patients with higher tumor load, we did not observe significantly decreased radiotracer accumulation in normal organs. Thus, we conclude that modifying the injected activity of the theranostic equivalent [^177^Lu]Lu/[^90^Y]Y-PentixaTher based on tumor burden is not necessary. This may also apply to dosing of “cold” CXCR4 inhibitors currently used as anti-cancer drugs, e.g., Plerixafor or Olaptesed pegol [9, 10]. A CXCR4-directed PET could then be scheduled prior to initiating treatment with those drugs, e.g., to visualize the target in vivo or to quantify undesired off-target effects in normal organs [24]. Drug doses, however, should not be altered based on the tumor burden.

In addition, relative to PSMA or SSTR which are overexpressed on the tumor cell surface [25, 26], CXCR4 is a dynamic receptor expressed mainly in the tumor microenvironment [27]. For instance, formation of heteromers of CXCR7 with CXCR4 may then modulate affinity [28]. Although such information is not available in the context of CXCR4-targeted endoradiotherapy, future studies investigating a tumor sink effect on [^68^Ga]Ga-PentixaFor PET should also consider potential receptor fluctuations prior to CXCR4-directed therapies, e.g., due to interaction of CXCR4 with other chemokine receptors on a subcellular level.

The missing tumor sink effect could also have an impact on scan interpretation, e.g., if CXCR4-expressing lesions are close to normal organs, such as spleen or bone marrow. Such a scenario may only occasionally occur, but can pose a challenge for less experienced readers. For other theranostic radiotracers, standardized frameworks for image interpretation have been proposed and validated [31, 32]. Thus, such a standardized assessment may also be helpful for interpreting [^68^Ga]Ga-PentixaFor PET/CT and could then increase reader’s confidence to classify lesions as benign or malignant, even when such lesions are close to normal organs with high tracer uptake.

We identified several limitations in this study. Although this is one of the largest cohort of solid tumor patients imaged with [^68^Ga]Ga-PentixaFor to date, the number of patients is still modest and may limit the ability to make definite conclusions. Therefore, future prospective studies should test the hypothesis of a tumor sink effect in a larger number of individuals. In addition, other factors should also be considered, e.g., the impact of concomitant therapies [30] or other day-to-day variables [33].

## CONCLUSIONS

In this study using [^68^Ga]Ga-PentixaFor in various solid tumors, we did not observe a substantial tumor sink effect. As such, injected activity of the theranostic equivalent [^177^Lu]Lu-/[^90^Y]Y-PentixaTher should not be modified based on the tumor burden. This may also apply to drug dosing of non-radiolabeled CXCR4-inhibitors used as anti-cancer agents.
